# The clinical significance of T-cell regulation in hypertension treatment

**DOI:** 10.3389/fimmu.2025.1550206

**Published:** 2025-02-26

**Authors:** Miaoxin Fu, Mingzhu Lv, Jinyue Guo, Aihua Mei, Hang Qian, Handong Yang, Wenwen Wu, Zhixin Liu, Jixin Zhong, Ying Wei, Xinwen Min, Haiyan Wu, Jun Chen

**Affiliations:** ^1^ Sinopharm Dongfeng General Hospital (Hubei Clinical Research Center of Hypertension), Hubei Key Laboratory of Wudang Local Chinese Medicine Research, Hubei University of Medicine, Shiyan, Hubei, China; ^2^ Shiyan Key Laboratory of Virology, Hubei University of Medicine, Shiyan, China; ^3^ School of Public Health, Hubei University of Medicine, Shiyan, Hubei, China; ^4^ Department of Rheumatology and Immunology, Tongji Hospital, Huazhong University of Science and Technology, Wuhan, Hubei, China

**Keywords:** hypertension, regulatory T cells, inflammation, immunotherapy, gene editing

## Abstract

Hypertension, a globally prevalent condition, is closely associated with T cell-mediated inflammatory responses. Studies have shown that T cells, by secreting pro-inflammatory cytokines such as interferon-gamma (IFN-γ), Interleukin-17 (IL-17), and Tumor necrosis factor-alpha (TNF-α), directly lead to vascular dysfunction and elevated blood pressure. The activation of Th1 and Th17 cell subsets, along with the dysfunction of regulatory T cells (Tregs), is a critical mechanism in the onset and progression of hypertension. This review explores the role of T cells in the pathophysiology of hypertension and discusses potential therapeutic strategies targeting T cell regulation, such as immunotherapy and gene-editing technologies. These emerging treatments hold promise for providing personalized therapeutic options for hypertensive patients, reducing inflammatory complications, and improving treatment outcomes.

## 1 Introduction

### 1.1 Background and importance

**Figure 1:**
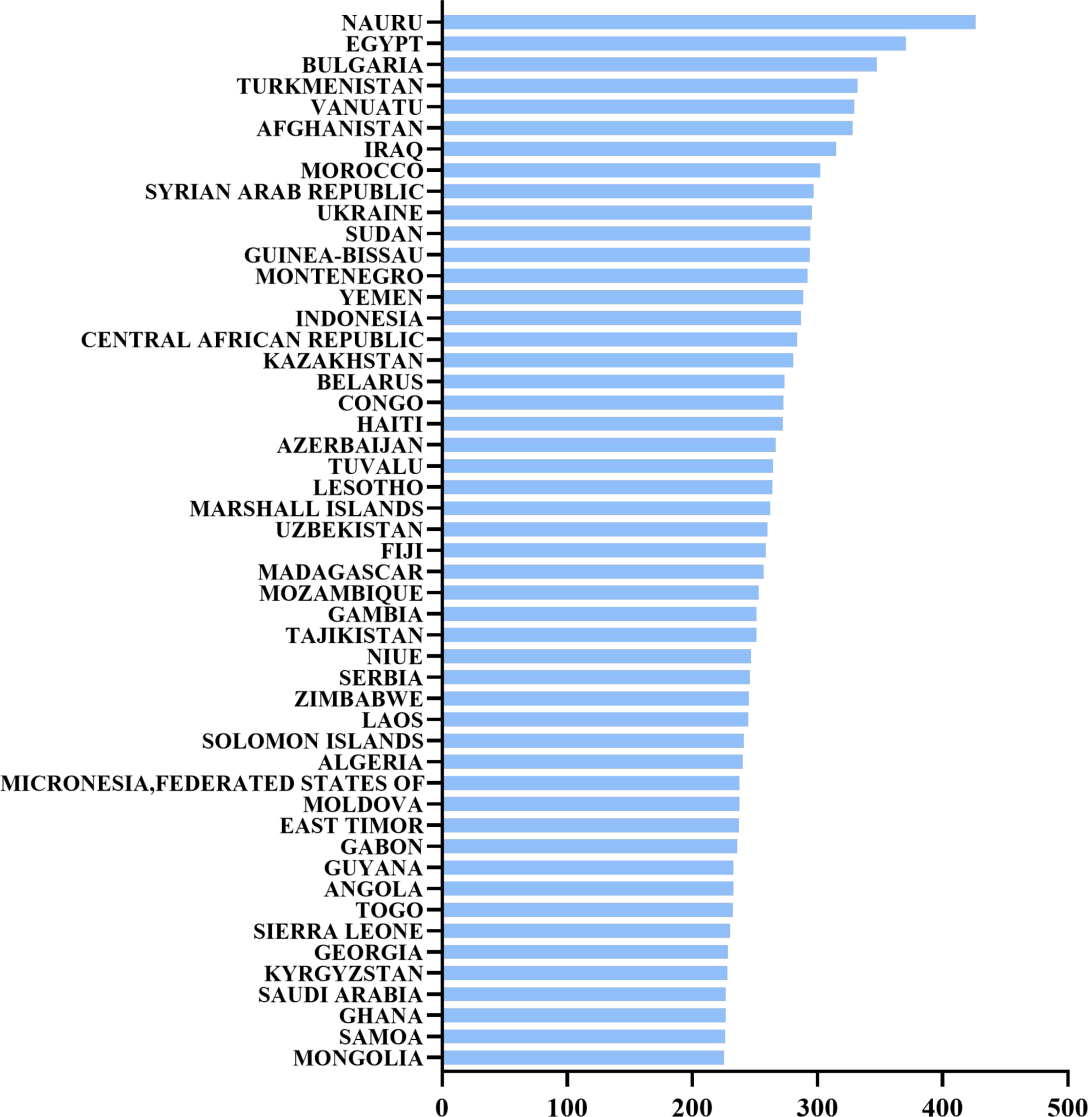
The world’s top 25 countries for hypertension mortality in 2021. Estimated annual death rate attributed to hypertension, also known as high blood pressure, per 100,000 people.

Hypertension is a globally prevalent cardiovascular disease and one of the leading causes of cardiovascular events and death. As shown in the chart below, the world’s top 25 countries for hypertension mortality in 2021 demonstrate a significant variation in the estimated annual death rates attributed to hypertension (1). This highlights the global burden of the disease and the disparities in hypertension management and control across different regions (**Figure 1**)

According to the latest data from the World Health Organization (WHO), over 1.1 billion adults worldwide have hypertension, with 78% residing in low- and middle-income countries, contributing to approximately 7.5 million deaths annually, accounting for 12.8% of global mortality. In high-income countries, the control rate of hypertension is 44%, while in low- and middle-income countries, it is only 21% (2, 3).In this context, numerous studies suggest that adopting stricter blood pressure classification standards, such as the new diagnostic threshold of 130/80 mmHg, could increase awareness of early screening and intervention (3). This new standard advances hypertension diagnosis earlier than the previously lenient threshold of 140/90 mmHg, aiming to prevent severe cardiovascular complications later in life through earlier lifestyle modifications and pharmacological interventions. Additionally, hypertension prevalence varies significantly across age, gender, and regions. Among younger adults (20-45 years), men have a higher prevalence of hypertension than women, whereas in the elderly population (65 years and older), the prevalence in women slightly surpasses that in men (4). The higher hypertension control rate in high-income countries contrasts with the lower rate in low- and middle-income countries, reflecting disparities in hypertension management globally (2). Hypertension not only poses a major risk for cardiovascular events but is also closely related to various organ system disorders, including stroke, myocardial infarction, and renal failure (3).

In recent years, growing evidence has highlighted the connection between the immune system and the pathogenesis of hypertension, particularly the crucial role of T cells in hypertension pathophysiology. T cells, traditionally recognized for their role in immune responses, directly participate in hypertension development by influencing vascular endothelial cells and triggering chronic inflammatory responses. Studies indicate that T cells, through the release of multiple cytokines and chemokines, can induce endothelial damage and vascular dysfunction, leading to elevated blood pressure (5–7). RANTES (Regulated on Activation, Normal T Cell Expressed and Secreted), an important chemokine, plays a pivotal role in the inflammatory response and T-cell accumulation associated with hypertension. By promoting T-cell migration to inflammatory sites, RANTES enhances local immune responses and exacerbates vascular dysfunction (8). This discovery provides new possibilities for immune-regulatory targets in hypertension treatment. In summary, modulating T-cell-mediated inflammatory responses and balancing T-cell subtypes could represent potential therapeutic targets for hypertension, promoting the development of personalized and innovative treatment strategies.

### 1.2 The clinical status of hypertension and its health impact

Hypertension is a common chronic disease with a complex pathogenesis. Long-term hypertension can lead to severe complications, particularly affecting the cardiovascular, renal, and ocular systems, posing significant threats to patient health and survival. According to WHO statistics, approximately 12.5 million cardiovascular disease-related deaths each year are associated with hypertension, including 3.5 million cases of myocardial infarction and 9.5 million strokes (2). Cardiovascular complications, such as stroke and myocardial infarction, are particularly prominent; about 54% of strokes are associated with hypertension, and myocardial infarction is the leading cause of death in cardiovascular patients, with hypertension increasing its risk by 2-3 times (3). Additionally, the incidence of chronic kidney disease (CKD) is significantly higher in hypertensive patients, with hypertension being the primary cause of 25-30% of end-stage renal disease (ESRD) (9). Hypertension can also cause retinal damage, impacting vision.

Despite the availability of multiple antihypertensive drugs, including ACE inhibitors, beta-blockers, and calcium channel blockers, drug resistance remains a major challenge. Studies indicate that 15-30% of hypertensive patients develop resistance to one or more antihypertensive drugs, leading to suboptimal blood pressure control (10). This resistance may be linked to genetic differences, metabolic abnormalities, and lifestyle factors such as high salt intake and lack of exercise (5). Furthermore, patient compliance is an issue, with about 50% of hypertensive patients failing to consistently adhere to prescribed medications due to side effects, adverse reactions, and insufficient understanding of the disease and treatment. To address this issue, regular blood pressure monitoring, comprehensive treatments combining medication and lifestyle interventions, and research into novel antihypertensive drugs and personalized treatment strategies are crucial. These measures can enhance hypertension management and improve patient quality of life (11).

## 2 Immunological basis of hypertension

### 2.1 Interaction between hypertension and the immune system

Hypertension is a chronic condition, and recent research suggests a complex interplay between hypertension and the immune system. Immune cells, which include lymphocytes (such as T cells and B cells), macrophages, and natural killer (NK) cells, are responsible for immune surveillance, defense, and homeostasis, playing key roles in infection defense and tissue repair. During the pathogenesis of hypertension, immune cell activation and functional alterations can trigger vascular inflammation and remodeling.

The interaction between macrophages and T cells is a critical regulatory mechanism. Macrophages contribute significantly to endothelial cell damage and inflammation by secreting pro-inflammatory cytokines like IL-6 and TNF-α, which activate T cells, thereby exacerbating vascular inflammation (12). This interaction depends on the macrophage’s functional phenotype, which ranges from pro-inflammatory M1 to anti-inflammatory M2 (13). This creates a vicious cycle wherein macrophage and T cell activation synergistically enhance inflammation, further damaging the endothelium and worsening hypertension. Activated T cells also release IFN-γ, which amplifies macrophage inflammatory responses, further damaging the endothelium and promoting vascular hardening, ultimately driving hypertension progression (14). Additionally, T cells interact with B cells through CD40 signaling, leading to the production of pro-inflammatory antibodies (especially IgG) by B cells, which enhance macrophage-driven inflammation (15–18). Meanwhile, B cells secrete IL-10 to modulate T cell function and maintain the balance of T cell subsets (19). For instance, B cells help maintain the balance between Th1 and Th17 cells, preventing the differentiation of naïve T cells into these pro-inflammatory subsets, while promoting Treg expansion to suppress pathological Th1/Th17 responses (20, 21). This interplay of immune cells highlights the potential for targeting T cell, B cell, and macrophage functions as novel therapeutic strategies for hypertension. By further understanding these interactions, researchers can explore new therapeutic targets for better managing hypertension and its complications.

### 2.2 Role of T cells in hypertension

T cells, as a subset of lymphocytes, play an essential role in the immune response and have garnered significant attention in the context of hypertension pathophysiology. T-cell-mediated inflammation contributes to the progression of hypertension, with different T cell subsets and their secreted cytokines playing crucial roles in both the onset and progression of the disease.

Th1 cells, for example, release pro-inflammatory cytokines such as IFN-γ, which are implicated in vascular smooth muscle cell (VSMC) proliferation and vascular inflammation. Recent studies have demonstrated that Th1 cells activate the angiotensin II (Ang II) signaling pathway, promoting VSMC proliferation and triggering vascular inflammation, which can elevate blood pressure (14). Th1-mediated inflammation contributes to the hypertensive pathological process through immune responses that promote vascular damage.

Similarly, Th17 cells secrete IL-17, which has been shown to play a significant role in Ang II-induced hypertension. IL-17 exacerbates vascular inflammation, increases vasoconstriction, and promotes endothelial dysfunction, directly driving the pathological processes of hypertension (6). Additionally, RANTES (Regulated upon Activation, Normal T cell Expressed and Secreted), a key chemokine, not only attracts T cells to inflammatory sites but also intensifies vascular inflammation, worsening the progression of hypertension (8).

The imbalance between T-cell subsets is another critical factor contributing to hypertension. A reduction in regulatory T cells (Tregs) weakens their inhibitory effect on pro-inflammatory T cells, such as Th1 and Th17 cells, leading to uncontrolled inflammation that exacerbates hypertension. This imbalance is particularly prominent in vascular stiffening, where a lack of Tregs leads to endothelial dysfunction and inflammatory dysregulation, further aggravating hypertension (22). Moreover, studies suggest that T cells can maintain immune balance in hypertension by regulating Th1 and Th17 cell activity, especially in vascular lesions where the immune balance is disturbed (23).

Recent research has also highlighted the importance of epigenetic modifications in regulating T-cell function, particularly in hypertension. Mechanisms such as DNA methylation and histone modifications alter the activation state of T cells, suggesting that gene regulation may contribute significantly to the role of T cells in hypertension beyond just inflammatory responses (24, 25). This highlights the complex regulatory network that influences T cell activity and its contribution to hypertension pathogenesis.

Pro-inflammatory cytokines, such as TNF-α, IL-6, and MCP-1, also play critical roles in T-cell mediated hypertension (26, 27). TNF-α is linked to chronic inflammation, promoting VSMC proliferation, vascular hardening, and the activation of the NF-κB pathway in T cells, further driving vascular damage (28–30). IL-6 and MCP-1, secreted by T cells, activate inflammatory signaling pathways such as the JAK/STAT pathway, contributing to the chronic inflammation that exacerbates hypertension (27, 31, 32).

Although these preclinical studies highlight the critical role of T cells in hypertension, relevant clinical trials are still ongoing. Further clinical data will help validate these findings and guide clinical treatment strategies.

## 3 Molecular mechanisms of T cell regulation

### 3.1 Signaling pathways of T cell activation and differentiation

T cell activation is the cornerstone of immune response, involving the proliferation and differentiation of T cells into effector cells. This process requires two signals: the first is delivered through the T-cell receptor (TCR) and is enhanced by adhesion molecules; the second is a co-stimulatory signal, provided by the interaction between co-stimulatory molecules on antigen-presenting cells (APCs) and receptors on T cells, which amplifies the TCR signal (33, 34). The first signal alone cannot induce a full immune response in T cells. Without the second signal, T cells enter a state of anergy, immune tolerance, or undergo programmed cell death, likely due to limited activation of the major histocompatibility complex (MHC)-peptide-TCR complex and internalization of the TCR-CD3 complex. Co-stimulatory complexes like B7-CD28 can prevent such outcomes (35). The first signal determines the specificity of T cell activation, while the co-stimulatory signal directs the functional outcome (36).

Different T cell subsets and their secreted cytokines regulate inflammation through multiple signaling pathways, which play critical roles in the pathogenesis of hypertension. T cell activation and differentiation not only rely on antigen recognition but also involve intricate signaling cascades, including the TCR, NF-κB, JAK/STAT, mTOR, and Notch pathways.

The TCR signaling pathway is central to T cell activation. Upon TCR binding to its antigen, downstream signals such as NF-κB and MAPK are triggered, promoting T cell proliferation and functional differentiation (33). TCR activation induces specific T cell subsets (e.g., Th1 cells) to secrete large amounts of pro-inflammatory cytokines, including IFN-γ and TNF-α (33, 37). These cytokines exacerbate immune responses in hypertension and, through various signaling pathways, stimulate vascular smooth muscle cell proliferation and vascular remodeling, leading to further elevation in blood pressure.

The NF-κB signaling pathway plays a crucial role in T cell-mediated inflammatory responses. Activated by TCR and cytokine signals, NF-κB regulates the expression of various pro-inflammatory cytokines, such as TNF-α and IL-6 (37). Sustained activation of the NF-κB pathway in chronic inflammation accelerates vascular damage, contributing to endothelial dysfunction and arterial stiffening (38–40). NF-κB signaling pathway, activated via Angiotensin II binding to the Angiotensin II type 1 receptor (AT1R), is involved in pro-inflammatory responses, promoting vascular smooth muscle cell proliferation and vascular remodeling, indirectly leading to elevated blood pressure (40–43). The JAK/STAT signaling pathway is closely related to T cell differentiation, particularly in the differentiation of Th17 cells (44). IL-6 activates the JAK/STAT3 pathway, inducing Th17 differentiation and promoting IL-17 secretion (31, 45–47). IL-17 amplifies vascular inflammation and endothelial dysfunction, exacerbating hypertension. Inhibiting the IL-6/JAK/STAT3 pathway reduces Th17 activity, alleviating vascular inflammation and damage in hypertension (6, 31, 48, 49). The mTOR pathway, which regulates T cell metabolism, influences T cell differentiation as well. In hypertension, excessive mTOR activation enhances the proliferation of pro-inflammatory T cell subsets, such as Th1 and Th17, aggravating inflammatory responses. Studies in animal models have shown that mTOR inhibitors can reduce inflammatory T cell numbers and lower blood pressure (50–52). The Notch signaling pathway also plays a pivotal role in T cell differentiation, particularly in regulating Th1 and Th17 differentiation (53). While Notch signaling is linked to pro-inflammatory T cell activation and its role in vascular inflammation, whether direct inhibition of Notch can significantly lower blood pressure in hypertensive patients remains insufficiently supported. Animal studies suggest that inhibiting Notch signaling can reduce inflammation, but its effect on lowering blood pressure alone has not been conclusively demonstrated.

**Figure 2:**
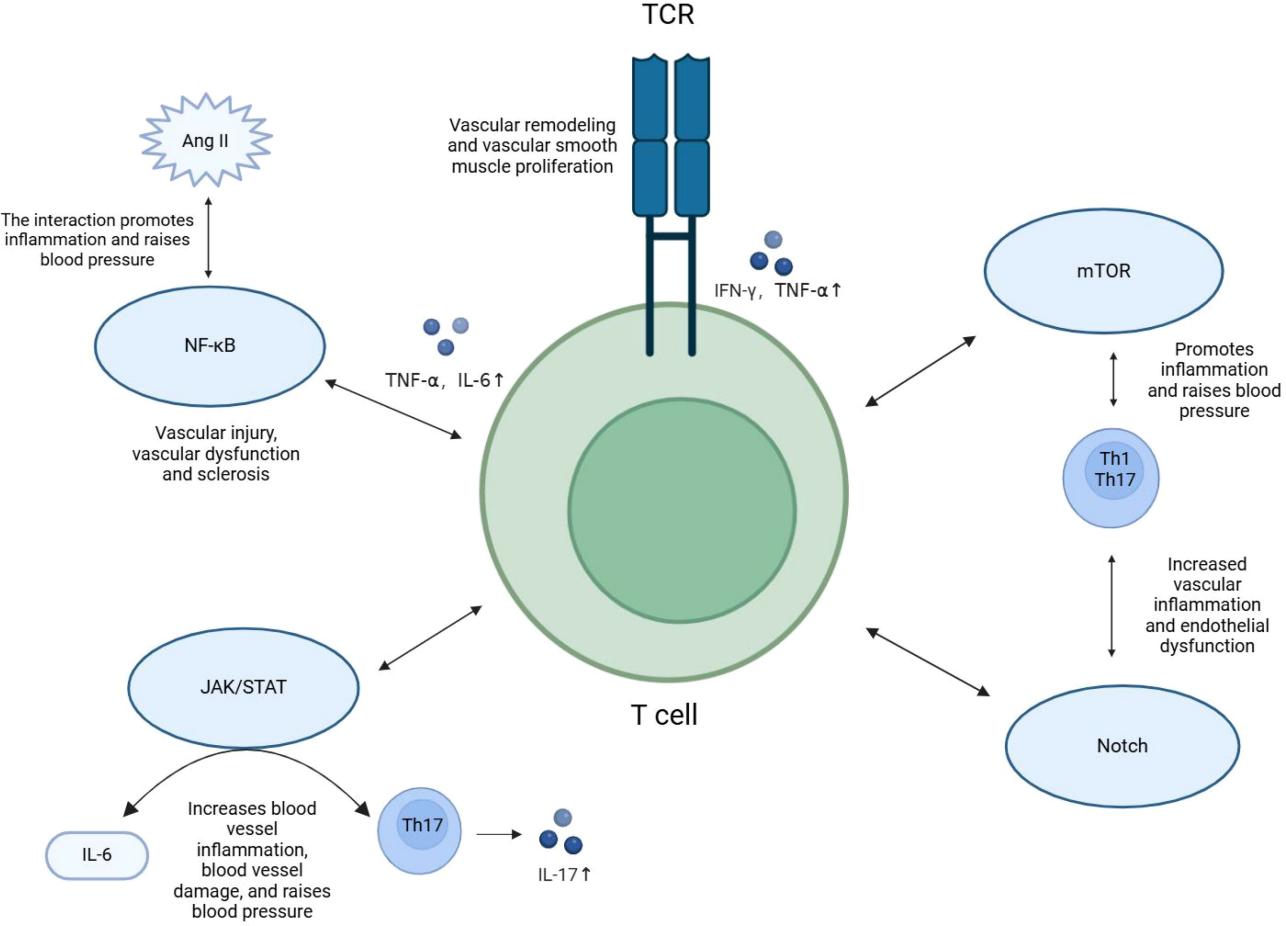
Signaling pathways for T cell activation and differentiation. T cell subsets and their secreted cytokines regulate inflammation through signaling pathways such as TCR, NF-kB, JAK/STAT, mTOR, and Notch, playing a role in the pathogenesis of hypertension.

In summary, T cell activation and differentiation signaling pathways regulate the expression of pro-inflammatory and anti-inflammatory cytokines, playing an important role in the pathophysiology of hypertension. Interventions targeting these signaling pathways offer new avenues for future immunotherapy in hypertension, particularly in reducing inflammation and controlling blood pressure (**Figure 2**)

### 3.2 Role of epigenetic and post-transcriptional modifications in T cell function

Recent studies have increasingly focused on the role of epigenetic and post-transcriptional modifications in regulating T cell function. These regulatory mechanisms are crucial not only for T cell activation, proliferation, differentiation, and maintenance of function but are also closely linked to the development and progression of inflammatory diseases like hypertension.

DNA methylation is a classic form of epigenetic regulation, catalyzed by DNA methyltransferases (DNMTs), which add methyl groups to CpG islands within genes, thereby modulating gene expression. In T cells, DNA methylation plays a key role in determining their differentiation and functional status (24). For instance, the Foxp3 gene, which is essential for the differentiation of regulatory T cells (Tregs), is influenced by its DNA methylation status (54). Elevated methylation of the Foxp3 gene in hypertensive patients weakens Treg function, exacerbating immune inflammation and vascular damage. Research has shown that reducing the methylation of Foxp3 can restore Treg function, potentially alleviating hypertension-associated immune inflammation (55–57).

Not only Tregs, but pro-inflammatory T cell subsets like Th1 and Th17 also rely on DNA methylation for their differentiation. DNMTs suppress excessive activation of these cells by methylating key genes (58–61). Studies have shown that inhibiting DNMT activity can significantly reduce the proliferation of Th1 and Th17 cells and suppress the secretion of pro-inflammatory cytokines, such as IFN-γ and IL-17, thereby alleviating chronic inflammation in hypertension (61). This mechanism presents new possibilities for controlling pro-inflammatory immune responses through epigenetic regulation.

MicroRNAs (miRNAs), as key post-transcriptional gene regulators, modulate T cell function by binding to target mRNAs, inhibiting their translation or inducing their degradation (62). miRNAs finely regulate T cell activation, differentiation, and cytokine secretion. For instance, miR-21 is upregulated in hypertensive patients and regulates Treg function, mitigating inflammation and improving vascular function (63–65). In contrast, miR-155 is highly expressed in pro-inflammatory T cells (e.g., Th1 and Th17 cells), enhancing their inflammatory activity and promoting the secretion of IFN-γ and IL-17, thereby aggravating vascular inflammation (66, 67). Targeting miR-155 may reduce the secretion of pro-inflammatory cytokines, alleviating hypertension-related vascular dysfunction (67, 68).

Notably, epigenetic and post-transcriptional modifications are not isolated processes; they interact to co-regulate T cell fate and function. For example, miRNAs can influence DNA methylation by regulating DNMT expression, while also modulating other epigenetic factors, thus extending the regulatory network of T cell functions (69). This interaction increases the complexity of T cell regulation and plays a key role in inflammatory diseases like hypertension.

In conclusion, epigenetic modifications (such as DNA methylation) and post-transcriptional modifications (such as miRNAs) regulate T cell differentiation and function through multiple layers and pathways, driving the immune pathology of hypertension. Future research can further explore these regulatory mechanisms as potential therapeutic targets in hypertension, with broad clinical applications in reducing vascular inflammation and improving vascular function.

### 3.3 T cells and hypertension-related molecular markers

Recent research has revealed that T cells and their secreted pro-inflammatory cytokines play a central role in the pathogenesis of hypertension. Th1 and Th17 cells, along with their products, such as IFN-γ, TNF-α, and IL-17, significantly contribute to vascular inflammation and dysfunction (70). For example, IL-17 induces local inflammatory responses by activating endothelial cells, eventually leading to vascular remodeling and hypertension. Clinical data have shown that IL-17 expression in hypertensive patients correlates positively with blood pressure levels and cardiovascular risk (6, 71). IFN-γ, by activating the Angiotensin II signaling pathway, promotes vascular smooth muscle cell proliferation and exacerbates inflammation, worsening the condition (72). TNF-α is closely linked to chronic inflammation and vascular stiffening, activating the NF-kB signaling pathway, which enhances T cell activation and the release of pro-inflammatory cytokines, accelerating vascular damage and remodeling associated with hypertension (73–75).

Targeted interventions against these molecular markers have become a focus in immunotherapy research for hypertension. For instance, IL-17 inhibitors have demonstrated significant therapeutic effects in animal models, not only reducing blood pressure but also mitigating vascular inflammatory damage (76, 77). Similarly, blocking IFN-γ and TNF-α signaling pathways can reduce vascular inflammation and remodeling, thereby lowering cardiovascular risk.

Furthermore, epigenetic regulation, such as the methylation level of the Foxp3 gene, directly affects the anti-inflammatory function of regulatory T cells (Tregs). Studies have shown that modulating the methylation state of the Foxp3 gene can enhance Tregs’ anti-inflammatory activity, potentially alleviating hypertension-related immune inflammation (78). These findings underscore the critical role of T cells in hypertension and provide promising directions for immunotherapy targeting T cell-related molecular markers, advancing personalized treatment strategies.

## 4 Clinical review of hypertension treatments

### 4.1 Effects of current hypertension treatments on T cells

#### 4.1.1 Pharmacological treatment

Current hypertension medications influence T cell function through various mechanisms, thus modulating immune responses related to inflammation. Common antihypertensive drugs (such as ACE inhibitors [ACEI] and angiotensin receptor blockers [ARBs]) inhibit Th1 and Th17 cell activity, reducing the secretion of pro-inflammatory cytokines like IFN-γ and IL-17, which significantly lowers vascular inflammation and improves blood pressure control (79–82). Calcium channel blockers (CCBs) and diuretics reduce Angiotensin II levels, indirectly inhibiting T cell activation and pro-inflammatory cytokine release, thereby preserving vascular function (83–85). Beta-blockers not only modulate regulatory T cell (Treg) function through metabolic and epigenetic pathways but also promote Treg activation, modulating immune responses and alleviating chronic inflammation in hypertension (86–88). Additionally, Tocilizumab, an anti-inflammatory drug targeting IL-6, is primarily used to treat immune-mediated inflammatory diseases like rheumatoid arthritis. While its mechanism of inhibiting Th17 cell activation and reducing inflammatory cytokines through the IL-6/JAK/STAT pathway has been validated in other diseases, its application in hypertension remains in the exploratory phase. Current research has yet to establish the widespread clinical use of Tocilizumab in managing immune inflammation in hypertension (89–92). Therefore, Tocilizumab’s potential role in hypertension warrants further investigation, though its immunomodulatory mechanism offers new possibilities for personalized hypertension treatment.

These studies highlight the potential of improving hypertension treatment outcomes by regulating T cell function, suggesting that immune modulation not only effectively controls blood pressure but also reduces hypertension-related inflammation and vascular damage.

#### 4.1.2 Lifestyle interventions

Lifestyle changes also play a significant role in regulating T cell-mediated inflammatory responses in non-pharmacological treatments. Research has shown that the Mediterranean diet, rich in polyunsaturated fatty acids and antioxidants, effectively inhibits pro-inflammatory T cell subsets (e.g., Th1, Th17), while increasing the proportion of regulatory T cells (Tregs), potentially reducing immune inflammation in hypertensive patients (93, 94). A clinical study demonstrated that the Mediterranean diet significantly lowered IL-6 and TNF-α levels in the blood, reducing the risk of developing hypertension (94–101). Additionally, low-sodium diets and regular aerobic exercise reduce Th17 cell activation and increase Treg proportions, significantly lowering blood pressure and reducing vascular inflammation (94, 102). Exercise interventions also have important benefits for improving T cell function. Regular aerobic exercise not only inhibits pro-inflammatory T cell activation and reduces IL-17 secretion but also promotes the recovery of Treg function, alleviating vascular inflammation and immune imbalance (103–108). Exercise also improves obesity-related metabolic disorders, indirectly modulating T cell activity, further enhancing vascular function and lowering blood pressure (109, 110).

Thus, lifestyle interventions provide an effective non-pharmacological treatment strategy for correcting T cell dysfunction, offering long-term vascular protection for hypertensive patients, and providing essential evidence for the development of personalized management plans.

### 4.2 T cell regulation in novel therapeutic strategies

#### 4.2.1 Gene therapy

Recent advancements in gene therapy technology have made significant progress in research on immune regulation related to hypertension. T cells, particularly regulatory T cells (Tregs), play a crucial role in the chronic inflammation associated with hypertension. Thus, restoring and enhancing Treg function has emerged as a promising new strategy in hypertension treatment. CRISPR/Cas9 gene-editing technology has shown great potential for modulating Treg function, particularly by targeting the expression of the Foxp3 gene to restore its immunosuppressive function (111, 112). Foxp3 acts as the central regulator of Treg function, and hypermethylation of Foxp3 is closely associated with Treg dysfunction (113, 114). By precisely regulating Foxp3 expression using CRISPR/Cas9 technology, it is possible to restore the anti-inflammatory properties of Tregs while also reducing the excessive activation of pro-inflammatory T cells, such as Th1 and Th17 cells, thus mitigating endothelial damage (111, 115, 116). Recent studies have further demonstrated that modulating the methylation status of the Foxp3 gene can enhance Treg-mediated immunoregulation, which in turn helps alleviate chronic inflammation in hypertension (114, 117–121).

Despite these promising findings in animal models, the clinical application of gene therapy for hypertension in humans still faces significant challenges. These include concerns over the long-term safety, specificity, and potential off-target effects of gene editing. Therefore, future research must focus on optimizing the precision of gene-editing tools like CRISPR/Cas9 and exploring their individualized application in hypertensive patients to ensure efficacy and safety under specific pathological conditions.

#### 4.2.2 Cell therapy

Cell therapy, particularly Treg (regulatory T cell)-based therapy, has shown great potential in the treatment of hypertension. Studies have demonstrated that the number and function of Tregs are significantly reduced in hypertensive patients, while the proportion of pro-inflammatory T cells (e.g., Th17 cells) is elevated, exacerbating inflammatory responses and subsequently increasing blood pressure (22, 122–124). Tregs play a key role in immune regulation by suppressing the release of pro-inflammatory cytokines such as IL-17 and IFN-γ, effectively alleviating hypertension-related chronic inflammation (125, 126). Therefore, expanding and reinfusing autologous Tregs to restore their immunosuppressive abilities has emerged as a promising strategy for improving hypertension treatment (122, 127).

Moreover, researchers are exploring the genetic modification of Tregs to further enhance their immunosuppressive function. For example, using CRISPR/Cas9 technology to modify key regulatory genes in Tregs, such as Foxp3, can significantly improve Treg function and stability. Studies have found that genetically modified Tregs can maintain a longer-lasting anti-inflammatory effect *in vivo*, enhancing their ability to control chronic inflammation caused by hypertension (113, 124).

As technology advances, the application of Treg cell therapy in the immunoregulatory treatment of hypertension holds even greater promise. Future research will focus on improving the stability and safety of Treg cell therapies, particularly in terms of clinical operability. With further basic research and clinical trials, Treg cell therapy could become an important therapeutic approach, offering personalized and effective treatment options for hypertensive patients.

## 5 Challenges and future research directions

Research on T cell regulation in hypertension faces several significant limitations. A major issue is the small sample sizes in many studies, which reduce the statistical power of the results and hinder a comprehensive understanding of hypertension and its immune mechanisms. Additionally, existing studies tend to focus on isolated T cell subsets or specific pro-inflammatory factors, often overlooking the broader, systemic mechanisms that contribute to the pathogenesis of hypertension. This narrow focus impedes a holistic understanding of how T cells influence hypertension at a molecular and physiological level.

Recent advancements in genetics and gene editing technologies, particularly CRISPR/Cas9, present promising opportunities to address these challenges (128). One notable development is the identification of phosphodiesterase 3A (PDE3A) gene mutations that enhance enzyme activity and are associated with hypertension with brachydactyly (HTNB) (129, 130). This discovery opens new avenues for targeted therapeutic interventions, emphasizing the critical role of genetics in understanding hypertension. However, it also highlights several hurdles. A primary challenge is the need for more *in vivo* models to confirm the involvement of mutated PDE3A in hypertension development. Existing animal models often fail to replicate the immune responses observed in humans, which limits their utility in fully understanding the genetic-immune interactions that underlie hypertension (128, 130).

A significant barrier in hypertension research lies in the inadequacy of current animal models to faithfully simulate human hypertension, particularly in terms of immune system involvement. While advanced technologies like CRISPR/Cas9 have been used to generate PDE3A-mutant animal models, these models do not yet fully capture the immune dynamics of human hypertension (129, 130). For example, while overexpression of PDE3A in smooth muscle cells leads to increased vascular resistance and hypertension, the interaction between these genetic alterations and immune cells—especially T cell subsets—remains poorly understood. Further investigation into this aspect is needed to explore how these mutations impact immune cell function and contribute to hypertension pathogenesis.

To address these challenges, future research should focus on developing more refined animal models that better simulate human immune dynamics, with particular emphasis on T cell regulation. The interaction between PDE3A mutations and immune cells such as T cells and macrophages is a critical area of research. Understanding how PDE3A mutations influence T cell activation and proliferation could reveal novel gene-targeted therapies for hypertension. These insights may also help identify new pathways for modulating immune responses to reduce vascular damage and improve blood pressure regulation (131).

While these findings provide a solid foundation for future therapeutic strategies, it is important to explore how T cell-related discoveries can be translated into clinical applications. For instance, identifying specific T cell subtypes and their functional alterations in hypertensive patients may guide patient stratification, risk prediction, and individualized treatment strategies, such as the use of immunomodulatory agents. Furthermore, integrating immune and genomic data may lead to more precise treatments, improving patient outcomes and reducing complications. By bridging basic research to clinical practice, we anticipate that personalized treatment strategies will become more precise and tailored, offering new insights into individualized therapy.

Collaboration between genetics, immunology, and cardiology is essential for advancing this field. By integrating gene editing technologies with immune modulation strategies, researchers can create more accurate models of human hypertension, which will provide a clearer picture of the disease’s underlying mechanisms. Such integrated research approaches could uncover novel signaling pathways and mechanisms through which PDE3A mutations influence vascular changes and contribute to hypertension (131).

In the long term, gene therapy and immunotherapy could provide exciting new treatment options for hypertension. Future research should aim to translate findings from animal models into clinical applications. This includes using gene editing technologies to precisely target PDE3A mutations or modulate immune responses to alleviate hypertension (129–131). Overcoming challenges related to the safety, efficiency, and long-term effects of gene therapy will be key to bringing these novel approaches into clinical practice.

In conclusion, while PDE3A mutations represent a promising therapeutic target for hypertension, much work remains to be done. The integration of genetic research and immune regulation, coupled with the development of more precise animal models, will be crucial for advancing our understanding of hypertension and improving treatment strategies.

## 6 Future outlook

In the future, precision medicine and personalized treatment will become increasingly important in the management of hypertension. With the rapid development of genomics and immunology, scientists are expected to gain deeper insights into the role of T cells in hypertension, enabling tailored treatment plans for individual patients. Specifically, genomics research can identify genetic variations related to hypertension, particularly those affecting T cell function. This will provide a crucial basis for developing more precise treatment strategies. Moreover, immune phenotyping can reveal changes in T cell subsets within patients, helping clinicians select appropriate immunomodulatory drugs and adjust dosages to optimize therapeutic outcomes.

In this context, interdisciplinary collaboration and technological innovation will be key drivers of progress. By integrating knowledge from fields such as cardiology, immunology, and genomics, researchers can develop novel immunomodulatory drugs and gene therapies aimed at improving the prognosis of hypertensive patients. Overall, future research will continue to explore the role of T cells in hypertension, promote the development of new immunotherapies, and strive for significant progress in improving patients’ quality of life.

## 7 Conclusion

In conclusion, hypertension remains a global health crisis with significant mortality rates (102, 132, 133).Recent studies highlight the critical role of T cells in hypertension pathogenesis, particularly in immune regulation and inflammatory responses. Dysregulation of Tregs and the imbalance with pro-inflammatory T cells are central to hypertension-induced inflammation (134). Targeted T cell regulation through immunomodulatory drugs, gene therapy, and cell therapy offers new therapeutic possibilities.

Future research should focus on the interactions between T cells and other immune cells, exploring new regulatory molecules and refining animal models. Integrating immune and genomic data could lead to more precise, personalized treatments, improving patient outcomes and reducing complications. T cell regulation holds great potential for advancing hypertension therapy, offering new insights into individualized treatment strategies.
